# A qualitative exploration of pharmacovigilance policy implementation in Jordan, Oman, and Kuwait using Matland’s ambiguity-conflict model

**DOI:** 10.1186/s12992-021-00751-y

**Published:** 2021-08-30

**Authors:** Hamza Y. Garashi, Douglas T. Steinke, Ellen I. Schafheutle

**Affiliations:** Division of Pharmacy and Optometry, School of Health Sciences, Faculty of Biology, Medicine and Health, The University of Manchester, M13 9PT Manchester, UK

**Keywords:** Pharmacovigilance, Adverse drug reactions, Policy implementation, Developing countries, Arab World

## Abstract

**Background:**

As Arab countries seek to implement the ‘Guideline on Good Pharmacovigilance Practice (GVP) for Arab countries’, understanding policy implementation mechanisms and the factors impacting it can inform best implementation practice. This study aimed to explore the mechanisms of and factors influencing pharmacovigilance policy implementation in Arab countries with more established pharmacovigilance systems (Jordan, Oman), to inform policy implementation in a country with a nascent pharmacovigilance system (Kuwait).

**Results:**

Matland’s ambiguity-conflict model served to frame data analysis from 56 face-to-face interviews, which showed that policy ambiguity and conflict were low in Jordan and Oman, suggesting an “administrative implementation” pathway. In Kuwait, policy ambiguity was high while sentiments about policy conflict were varied, suggesting a mixture between “experimental implementation” and “symbolic implementation”. Factors reducing policy ambiguity in Jordan and Oman included: decision-makers’ guidance to implementors, stakeholder involvement in the policy’s development and implementation, training of policy implementors throughout the implementation process, clearly outlined policy goals and means, and presence of a strategic implementation plan with appropriate timelines as well as a monitoring mechanism. In contrast, policy ambiguity in Kuwait stemmed from the absence or lack of attention to these factors. Factors reducing policy conflict included: the policy’s compliance with internationally recognised standards and the policy’s fit with local capabilities (all three countries), decision-makers’ cooperation with and support of the national centre as well as stakeholders’ agreement on policy goals and means (Jordan and Oman) and adopting a stepwise approach to implementation (Jordan).

**Conclusions:**

Using Matland’s model, both the mechanism of and factors impacting successful pharmacovigilance policy implementation were identified. This informed recommendations for best implementation practice in Arab as well as other countries with nascent pharmacovigilance systems, including increased managerial engagement and support, greater stakeholder involvement in policy development and implementation, and undertaking more detailed implementation planning.

## Background

Since the thalidomide tragedy in the 1960s, adverse drug reactions (ADRs) have garnered increased attention internationally, accompanied by a worrying upward trend in ADRs resulting from prescribed drugs. [1] Of particular relevance are ADRs which are unexpected or severe, leading to increased morbidity, mortality and financial loss, yet are often not recognised or identified before regulatory approval due to the limitations of clinical trials. [2, 3] The steady increase in medicine use worldwide is likely to increase the incidence of ADRs [1, 3].

To preserve public health and maintain confidence in the healthcare system, national governments implement policies in the form of a pharmacovigilance system to ensure the quality, safety, and effectiveness of approved drugs [4] according to the World Health Organization’s (WHO) guidelines [5–7]. Pharmacovigilance systems include mechanisms to monitor and evaluate drug safety throughout a medicine’s entire lifecycle. The system serves to collect and analyse reports of drug-related problems including ADRs by employing measures of quality control and assurance as well as disseminating information on potential risks to healthcare professionals and the public. [3]

At present, most of the world’s drug safety data originate from the developed world. [8, 9] However, differences in local factors including drugs’ effects on patients, prescribing patterns, regulation methods, quality, and availability mean that data used in assessing ADRs may have limited validity or relevance for patients living outside these countries. [10, 11] Hence, countries outside the developed world must implement policies aimed at building national pharmacovigilance systems. [12]

Globally, there is great disparity among countries in pharmacovigilance systems development and implementation. [13–15] Most developed countries have well-established systems, whereas many developing countries still lack fully functioning systems. [16, 17] The challenges many developing countries face are ADR under-reporting, human resource shortages, inadequate financial resources, as well as poor policy and legal framework. [9, 10, 17, 18]

The Arab World is experiencing significant activity with regards to pharmacovigilance. [19] However, like other developing countries, Arab countries differ significantly in their pharmacovigilance systems’ development level, ranging from those having either weak or non-existent systems (e.g. Kuwait, Djibouti, Lebanon, Palestine, and Qatar), to others having systems comparable to those in developed countries (e.g. Morocco, Egypt, Jordan, and Saudi Arabia). [11, 20–22] To harmonise pharmacovigilance practices in the Arab World, the Arab League developed the ‘Guideline on Good Pharmacovigilance Practices (GVP) for Arab Countries’, which is based on the European GVP guideline and was due for implementation by July 2015. [23]

As Arab countries seek to implement the Arab GVP guideline; and given pharmacovigilance’s importance as part of a country’s public health policies’ portfolio, understanding the mechanism(s) of policy implementation and the factors influencing it can inform best practice in nascent systems in the region. International experience has demonstrated that adopted policies are not always implemented as expected and do not necessarily achieve their intended results. [24, 25] Moreover, policymakers frequently focus on outputs or outcomes while ignoring the implementation process which could reveal the barriers to effective implementation. [26] Therefore, learning about the implementation process can assist in gaining a better understanding of the factors impacting policies’ success or failure. [27]

This paper aims to compare the mechanisms and factors influencing pharmacovigilance policy implementation in Arab countries with established systems to inform pharmacovigilance policy implementation in a country with a nascent system.

## Methods

### Theoretical framework

‘Policy implementation’ is defined as “the mechanisms, resources, and relationships that link policies to programme action” ([26], p. 37). This study adopted Matland’s [28] “ambiguity-conflict model” of policy implementation as a theoretical framework that synthesises many of the key insights from previous research on policy implementation. [29, 30] The model has been widely used in policy implementation analysis, the description and analysis of the relationships between policy and practice, implementation success or failure, and has produced valuable insights regarding policy implementation. [31] The model considers the extent of conflict associated with a policy and the level of ambiguity in policy development and implementation as the key factors shaping the implementation process. The extent of policy ambiguity and conflict are used to explain how different approaches to policy implementation occur. Matland [28] characterises policy conflict as the situation where “more than one organisation sees a policy as directly relevant to its interests and when the organisations have incongruous views” ([28], p. 156 ). Policy conflict increases the difficulty of successful policy implementation. Policy actors’ perceptions of ambiguity in goals and means increase the impact of contextual factors. The framework combines these two dimensions into a four-cell matrix (illustrated in Table 1), with each cell representing a distinct approach to implementation with a central principle determining its outcomes. Despite the model’s presentation of ambiguity and conflict as dichotomous, Matland [28] stresses that the theoretical constructs are continuous, and that there is no tipping point causing radical shifts from one implementation type to another.

**Table 1:** Ambiguity-Conflict matrix: Policy implementation processes ([28, 32] p. 230)

	Low Conflict	High Conflict
**Low Ambiguity**	**Administrative Implementation**	**Political Implementation**
	• Goals are given and a means for problem-solving is known • A central authority has the information, resources, and sanction capability to enact the desired policy • Implementation is hierarchically ordered with each link receiving orders from the level above • The policy is spelt out explicitly at each level and there is agreement on responsibilities and tasks • Relatively uniform outcomes at the micro-level across many sites	• There is conflict over both goals and means • The implementation process is a key arena for conflict • Implementation outcomes are determined by the distribution of power • Compliance is not automatically forthcoming • Low ambiguity ensures that monitoring of compliance is relatively easy
**High Ambiguity**	**Experimental Implementation**	**Symbolic Implementation**
	• Outcomes depend largely on which actors are involved • Variation in outcomes from site to site • Outcomes are hard to predict • Opportunities for local entrepreneurs to create local policies • Compliance monitoring mechanisms are of limited relevance • The policy may become a low priority	• Ostensibly implausible combination • Salient symbols can produce high levels of conflict even when the policy is vague • Outcomes will vary across sites • Outcomes will depend upon the balance of local coalition strength • Policy ambiguity makes it difficult to monitor activities

### Study design

This was a qualitative, semi-structured interview study of policy implementors from the national pharmacovigilance centres and the pharmaceutical industry in three Arab countries. The current situation of pharmacovigilance policy implementation in Jordan, Oman, and Kuwait highlights the differences in the level of pharmacovigilance systems’ development. Although the three countries have all adopted the GVP for Arab countries as part of their national pharmacovigilance policies, they differ in several aspects that are summarised in Table 2. For example, despite all three countries’ national drug authorities being responsible for carrying out pharmacovigilance-related activities, Kuwait is the only country in which these activities are not being carried out within an independent department. Moreover, only in Jordan is the policy applied in the form of a law.

**Table 2:** Pharmacovigilance policy implementation situation in Jordan, Oman, and Kuwait. Adapted from Alshammari et al. [21]

	Jordan	Oman	Kuwait
**Organization overseeing pharmacovigilance**	Jordan Food and Drug Administration (JFDA)	Oman Ministry of Health’s Directorate General of Pharmaceutical Affairs and Drug Control (DGPA&DC)	Kuwait Ministry of Health’s Drug and Food Control Administration (KDFCA)
**Pharmacovigilance system structure**	National & Regional Centres	National Centre with Regional Centre Network	National Centre (Unofficial)
**Pharmacovigilance centre, department, or unit name**	Department of Rational Drug Use and Pharmacovigilance	Department of Pharmacovigilance and Drug Information	Drug Registration Department’s Quality Assurance Unit (Unofficial)
**National pharmacovigilance system** **establishment year**	2001	1992	2008
**Year joined WHO Program for International Drug Monitoring as full member**	2001	1995	2021
**Pharmacovigilance guidance or legislation**	Law titled “The Pharmacovigilance Directives”	Guidelines titled “Guideline on Good Pharmacovigilance Practices in Oman”	A memo issued by KDFCA to companies
**Dedicated budget**	No	No	No
**Number of staff members**	5 full-time employees	5 full-time employees	5 full-time employees, plus one part-time employee
**National adverse drug reaction (ADR) or pharmacovigilance advisory committee**	Health Hazard Evaluation Committee	No	No
**Standard adverse drug reaction reporting form present**	Yes	Yes	Yes
**Product types covered**	Pharmaceuticals, herbal medicines, cosmetics, biologicals, medical devices	Pharmaceuticals, herbal medicines, biologicals	Pharmaceuticals, herbal medicines, biologicals, medical devices
**Type of drug-related** **problems covered**	Suspected ADRs, lack of efficacy, quality defects, drug abuse/misuse, medication errors	Suspected ADRs, lack of efficacy, quality defects, medication errors, counterfeit	Suspected ADRs, lack of efficacy, quality defects, medication errors, drug abuse/misuse, counterfeit
**Computerized case-report management system**	Yes	Yes	No
**Qualified Person for Pharmacovigilance (QPPV) Requirement**	Yes	Yes	No
**Submission of regular** **Periodic Safety Update Reports (PSUR)/Periodic Benefit-Risk Evaluation Reports (PBRER)**	Originator and generic products	Originator products only	Originator and generic products
**Submission of Risk** **Management Plans (RMPs)**	Yes	Yes	Yes

Kuwait is one of the Arab countries without an officially recognised pharmacovigilance system [11, 20, 22], and whose performance lags behind many other Arab countries’ systems [20]. Therefore, it represents a country with a nascent pharmacovigilance system. Jordan and Oman were selected as two countries at more advanced levels of performance and implementation of the GVP for Arab countries. [11, 20] Jordan possessed a pharmacovigilance system which is among the highest performing systems in the region. [20] Oman’s pharmacovigilance system’s performance level falls in the middle between Kuwait and Jordan [20] and highlights the progression of pharmacovigilance policy implementation among Arab countries with pharmacovigilance systems at different levels of performance.

### Sampling and recruitment

Approval was sought from the three national drug authorities (Jordan Food and Drug Administration (JFDA), Oman’s Directorate General of Pharmaceutical Affairs and Drug Control (DGPA&DC) and Kuwait Drug and Food Control Administration (KDFCA)), as well as members of the pharmaceutical industry. University ethics approval was obtained. Eligible participants included individuals who were current or immediate past employees of the national drug authority with direct involvement in implementing the pharmacovigilance policy and individuals employed in a pharmaceutical company operating in the study country as the designated person responsible for pharmacovigilance. Sampling was purposive and employed snowballing. All potential participants were emailed a study information sheet by a gatekeeper at the national drug authority and asked to contact the researcher to participate.

Taking into consideration the type and diversity of the target study population, it was deemed sufficient to interview 12 to 20 individuals per country to achieve data saturation. [33, 34]

### Data collection

Following written consent, the primary author conducted, and audio recorded face-to-face interviews (in English) lasting 60 min on average. Extensive field notes were taken, and participants also completed a questionnaire capturing characteristics such as gender, educational background, and employment sector. Interviews were conducted sequentially from April through December 2019, starting in Jordan, followed by Oman, and finally Kuwait [20]. This sequential approach enabled the use of the insights gained from earlier in later interviews, and particularly to sense check potential recommendations in the least developed country with regards to their pharmacovigilance systems.

The interview topic guide was informed by Matland’s [28] ambiguity-conflict model and existing literature on policy implementation research [35–37]. To establish the type of policy implementation process followed in the study countries, the interviews focused on exploring the levels of and factors impacting policy ambiguity and conflict during the policy implementation process. This was achieved by identifying the facilitators and barriers to policy implementation, as well as participants’ perceptions regarding ambiguity and conflict through enquiring about their understanding and acceptance of its goals and means. The main questions covered in interviews are listed in Table 3.

**Table 3:** Key questions asked in interview topic guide

• What difficulties did you face in the implementation of your country’s pharmacovigilance policy?
• What issues assisted in the implementation of your country’s pharmacovigilance policy?
• How clear do you feel are your country’s pharmacovigilance policy goals?
• How clear is your country’s pharmacovigilance policy in describing how it is to be implemented?
• Is there any aspect of your country’s pharmacovigilance policy that you don’t agree with?

### Data analysis

Verbatim interview transcripts and field notes were managed using NVivo 12 and subjected to thematic framework analysis involving five steps: familiarisation, coding, thematic framework identification, charting data into a matrix, and data interpretation. [38] The analysis employed both an inductive and deductive approach to develop themes that provided rich and detailed descriptions of the dataset whilst mapping onto Matland’s [28] ambiguity-conflict model. Connections within the themes were made and key similarities and differences between countries as well as between participants from the two sectors were identified.

## Results

Fifty-six participants were interviewed (17 participants in Jordan, 16 in Oman, and 23 in Kuwait). All but two interviews were audio-recorded where only detailed notes were taken. All members of the national pharmacovigilance centres in the three countries (n = 5 per country) participated in the study along with an additional two members of the regional pharmacovigilance centres in Jordan. Most participants were pharmacists (n = 48) and mainly employed by the pharmaceutical industry (n = 38).

The findings of the study are presented in the form of a comparison between the three countries divided into two parts. The first part covers the two dimensions of Matland’s [28] model, namely the levels of ambiguity and conflict associated with the pharmacovigilance policy present in Jordan, Oman, and Kuwait. The second part covers the factors that impacted both policy ambiguity and conflict. To illustrate the extent of each country’s participants’ agreement surrounding these issues, the terms few (n ≤ 4 participants), some (n = 5–8 participants), many (n = 9–12 participants), and most (n ≥ 13 participants) are used.

### Participants’ perceptions of policy ambiguity and conflict

**Fig. 1:**
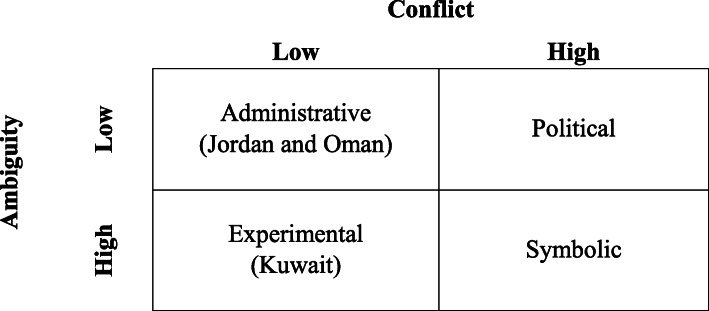
Study countries’ position on the ambiguity-conflict matrix based on perceptions of policy ambiguity and conflict. Adapted from Matland [28].

To assess ambiguity and conflict levels of the policy’s goals and means in the three countries, participants’ opinions on the extent of clarity of the country’s PV policy and its means of implementation were sought. Regarding conflict, participants’ views on whether they agreed with the policy’s goals and its method of implementation were solicited. Figure 1 reflects the position of each country on the ambiguity-conflict matrix based on participants’ perceptions of their country’s policy ambiguity and conflict.

#### Perceptions concerning policy ambiguity

Overall, participants’ responses pointed to low levels of perceived policy ambiguity in Jordan and Oman. National pharmacovigilance centre members in the two countries unanimously described their policies’ goals and means as clear. Many industry participants in Jordan agreed with this view, however, Oman’s industry participants had mixed views with some believing that the policy was ambiguous.

*“It’s [the policy] clear, it’s easy to understand, and if you have any questions, you can find it.“* (Participant 2, JFDA, Jordan).

*“They [the national centre] still have to clearly define what they actually want from others and what they are actually going to implement… little more clear statements and definitions should be given from the Ministry, the authority who’s implementing.“* (Participant 4, pharmaceutical industry, Oman).

Participants’ responses in Kuwait indicated that perceived policy ambiguity levels were high overall. While members of the national centre all agreed that the policy’s goals and means were clear, many industry participants had the opposite view.

*“…when it comes to implementing the [pharmacovigilance] system, still there is no clear guidance or clear regulation regarding this…”* (Participant 18, pharmaceutical industry, Kuwait).

#### Perceptions concerning policy conflict

Participants’ responses pointed to policy conflict levels being low overall in the three study countries. National pharmacovigilance centre participants in each country were all in agreement with their policies’ goals and means. Furthermore, many industry participants in Jordan and Oman, as well as most in Kuwait indicated the absence of policy conflict.

*“I agree [with the pharmacovigilance policy] because I’m able to perform the tasks that are requested.“ (Participant 8, pharmaceutical industry, Jordan)*.

*“I agree with the goals of the policy. I have no objection towards them. The policy is simple.“ (Participant 1, DGPA&DC, Oman)*.

### Factors impacting policy ambiguity and conflict

The themes extracted from the interviews are presented whilst identifying which group and which country they came from to allow for the comparison of the similarities and differences between them. Emerging themes were mapped onto Matland’s [28] model to identify the process and factors associated with successful pharmacovigilance policy implementation in the study countries.

The main themes extracted from the interviews were: political support, stakeholder involvement, training, policy characteristics, implementation planning, and pharmaceutical company-related factors. In what follows, the impact of the underlying factors pertaining to each theme will be first discussed as related to policy ambiguity followed by its impact on policy conflict. Table 4 summarises the results for the three study countries.

#### Political support

Participants from the national and regional centres in Jordan all agreed that decision-maker (political) support was a contributing factor to reducing policy ambiguity. This view was also held by a few members of the pharmaceutical industry in Jordan and Oman. Participants outlined how decision-makers engaged and communicated with policy implementors throughout the entire implementation process to minimise policy ambiguity by ensuring that there was an understanding of how it was to be carried out. Participants also identified decision-makers’ role in providing implementors with continuous support and encouragement which made them feel valued and increased confidence levels which helped in increasing the efficiency of the implementation process.

*“I feel that the administration was constantly supporting us. …I never got the impression that what we [members of the National Pharmacovigilance Centre] were doing was underestimated, and they [the administration] would discuss things with us such as why certain things were done. There was an understanding.”* (Participant 1, JFDA, Jordan).

In contrast, in Kuwait, a few national centre participants and some from the industry pointed to decision-makers’ (both within the Ministry of Health (MOH) and KDFCA) lack of encouragement or support as a barrier contributing to increased ambiguity. Moreover, pharmacovigilance policy implementation was reported by a few national centre and industry participants as the result of the personal endeavours of some of the staff at KDFCA. This lack of support resulted from what a few participants representing both sectors believed to be a lack of awareness of the subject of pharmacovigilance and thus the minimisation of its importance.

*“People think it’s not a serious thing. I’m not supposed to mention names or something, [but] there are people in charge who think what we do is very simple.”* (Participant 1, KDFCA, Kuwait).

Political (managerial) support was cited by a few participants from Jordan’s national and regional centres, as well as a few pharmaceutical industry participants from Jordan and Oman as a facilitator as it reduced the conflict in policy implementation means. This was evident in providing the national centre with official recognition, independence within the national drug authority’s organisational structure, and binding the policy to the law (in Jordan). This provided the national centre with the necessary legitimacy, importance, and authority to be able to carry out the implementation process. This further expedited obtaining approvals for conducting activities including training workshops, awareness campaigns, and conferences.

*“In terms of [facilitators] within the Directorate, there is the support of the administration… The support of the administration is demonstrated in the way they provide us with resources, or how they help in developing our skills, the planning, how they send us to courses or workshops related to pharmacovigilance. Another example is the director, who tries to teach us new things, keeping us updated with the latest information… So, there is communication.“* (Participant 10, DGPA&DC, Oman).

In Kuwait, MOH decision-makers’ lack of political will towards issues related to pharmacovigilance acted as a barrier to policy implementation according to a few participants from the national centre and some from the pharmaceutical industry. Participants believed that this stemmed from decision-makers’ resistance to (or fear of) change. Examples of this included the lack of official recognition of the national pharmacovigilance centre or the lack of a statutory provision for pharmacovigilance. This pointed to the presence of some conflict between implementors and decision-makers surrounding the policy means. The belief among these participants was that this was in part due to these decision-makers’ lack of understanding of pharmacovigilance’s importance.

*“The barriers of the implementation…the key personnel, the key personnel don’t know anything about pharmacovigilance. So that’s why I’m assuming that they will not implement such or they will not recognise such a guideline or such a mandate because of the knowledge, their knowledge. They don’t know what pharmacovigilance is.“* (Participant 18, pharmaceutical industry, Kuwait).

#### Stakeholder involvement

Many participants from Jordan’s pharmaceutical industry and a few from Oman’s described the collaboration between the national centre and other stakeholders during the policy development process as a facilitator. Participants described the examples of the pharmaceutical industry in Jordan and Oman being allowed to review and provide feedback on the policy during the drafting process, or other key stakeholders, such as the University of Jordan, or other departments in Oman's DGPA&DC being involved in the policy’s development. This participation had an important role in facilitating implementation through defining clear policy goals and thus reducing policy ambiguity and increasing consistency in implementation among the various stakeholders.

*“In the beginning, the JFDA prepared a draft guideline, and several companies received a copy of the draft guideline and they asked us to give our opinion and if we had any comments, which we did, and they took some comments into consideration until the final guideline was published.“* (Participant 14, pharmaceutical industry, Jordan).

Most participants in Kuwait (representing both sectors) were either unaware of or revealed that the pharmaceutical industry did not play a role in the policy development process. Participants described how the policy was issued as a memo from KDFCA without any prior involvement from the industry, with a few national centre participants revealing that the industry’s feedback was only obtained after the policy had been issued. This acted as a barrier because it led to some implementors from the pharmaceutical industry viewing the policy as ambiguous due to their lack of understanding of how the policy was to be implemented (i.e., policy means).

*“[A barrier to policy implementation was] The companies not understanding the guidelines clearly.“* (Participant 21, pharmaceutical industry, Kuwait).

A few participants from each of Jordan’s national centre and the pharmaceutical industry also outlined the importance of collaboration between both sides in the competency building of the country’s healthcare professionals in developing a robust pharmacovigilance system. This helped increase awareness levels and led to a reduction in policy ambiguity as illustrated by participants’ observations of increasing yearly ADR reporting rates.

*“So, it was not only the Health Authority wanting to implement the guideline. It was done hand in hand with the marketing authorisation holders, applicants, experts, and expert working groups. There were effective communication channels, effective competency building, and all groups working hand in hand. This was the major contributor to successful implementation.“* (Participant 5, pharmaceutical industry, Jordan).

Some participants from Jordan, many from Kuwait (almost equally represented by both sectors in the two countries), and a few from Oman (the majority from the pharmaceutical industry) shared the belief that, despite their efforts, healthcare professionals’, the public’s, and pharmaceutical company managers’ lack of knowledge, awareness, and/or understanding of the pharmacovigilance policy remained a major obstacle to implementation. They believed that these issues stemmed from these stakeholders’ perceived ambiguity regarding the goals and/or the means of implementing the policy. As such, both the national centre and the industry still struggled with ADR under-reporting.

*“… they [healthcare professionals] don’t know how to report or they don’t know the importance of reporting. They’re saying they don’t know where to report, how to report, what they have to do if this is the case…”* (Participant 7, pharmaceutical industry, Kuwait).

Like policy ambiguity, many industry participants from Jordan and a few from Oman indicated the importance of stakeholder involvement in the policy development process as a facilitator for its implementation by reducing policy conflict. Stakeholder involvement meant that agreement could be reached between all parties concerning its goals and means, which contributed to a reduction in resistance and subsequent delays in implementation.

*“…we [members of the national pharmacovigilance centre] discussed the subject [the pharmacovigilance policy] with the marketing authorisation [holders] and the pharmaceutical companies [and] it was well accepted. And because whatever we mentioned in our guideline, it was discussed with them and agreed with them.“* (Participant 7, DGPA&DC, Oman).

In terms of policy conflict, a lack of stakeholder involvement was an obstacle to implementation. A few participants from the national centre in each of the three countries and from the Jordanian regional centres as well as some industry participants in both Jordan and Kuwait shared how this lack of involvement was connected to healthcare professionals’ negative attitudes (or resistance) towards implementing the policy. Similarly, a few national centre participants in Oman and Kuwait and a few pharmaceutical industry participants in all three countries believed this negative attitude also existed among pharmaceutical company managers. In these cases, the policy was not viewed as a priority, but instead as an added burden that these stakeholder groups were forced to take upon themselves (i.e., conflict on policy goals and means). This lack of involvement was viewed as a contributing factor to the under-reporting of ADRs in the three countries.

*“Maybe [one of] the barriers, [is] the company. Maybe in the beginning they were not very aware of the importance of pharmacovigilance in the companies. Because it’s not stopping registration or marketing of any product. So, for the companies, it was not a priority to have a pharmacovigilance team in their companies.“* (Participant 8, pharmaceutical industry, Jordan).

#### Training

Some industry participants in Jordan and a few in Oman cited the training provided by members of the national centre in these countries to the industry as a facilitating factor, which made expectations clear and thus reduced policy ambiguity. This training was also recognised as helping to increase pharmacovigilance awareness, which in turn helped policy implementors develop a better understanding of the policy’s goals and its means of implementation.

*“…the authority had several workshops teaching the companies how to prepare a PSMF [pharmacovigilance system master file], a PSUR [periodic safety update report]. I think that also they had several workshops for healthcare professionals about pharmacovigilance, its importance, how to implement it, they tried to help people to some extent.“* (Participant 14, pharmaceutical industry, Jordan).

Unlike in Jordan and Oman, a few industry participants in Kuwait indicated that implementors from the pharmaceutical industry did not receive training regarding the policy. This led to a lack of knowledge which acted as a barrier and resulted in an implementation delay.

*“We need to know from where to start, for example, where does the reporting cycle start? We see all this on paper, but we haven’t yet actually implemented this into practice, nor do we know how to implement it. When they [the authority] issue guidelines they should be cooperative with us so that we can understand and implement.“* (Participant 5, pharmaceutical industry, Kuwait).

A perceived implementation barrier cited by a few national centre participants in Jordan and Oman was the lack of experience in pharmacovigilance of many of the centres’ employees. This resulted in perceived policy ambiguity among some implementors who considered it to be the duty of members of the national centre to explain and provide training on policy implementation.

*“… as a department it’s only been working since 2014 so we’re not talking about a long time. So, most of us [national pharmacovigilance centre staff] do not have that much experience. So, the low experience may be a reason [for difficulties in implementation]”* (Participant 2, JFDA, Jordan).

Interestingly, none of the participants cited training as a factor impacting policy conflict.

#### Policy characteristics

Participants in all three countries pointed to the nature of the policy itself as a facilitating factor for policy implementation. A few members each from the national centre and the pharmaceutical industry in Jordan and Oman pointed out that the policy was written in a manner that made it easier for implementors to understand its purpose and means of implementation. It was also emphasised that the level of detail present within the policy helped reduce ambiguity. The Jordanian JFDA’s creation of checklists to accompany the policy simplified implementation, and in Oman, this was achieved through the creation of a national abridged version of the original Arab GVP guideline.

*“…when we [members of the national pharmacovigilance centre] developed our own summarised guidelines, this process [implementation] became easier; we were able to communicate properly with the companies. We were able to understand the companies and they were able to understand us.“* (Participant 1, DGPA&DC, Oman).

*“It [the policy] is a simplified version of the Arab GVP guidelines. The companies have informed [us] that it is quite to the point and simple.“* (Participant 14, pharmaceutical industry, Oman).

Some industry participants from Kuwait noted a lack of clarity and detail (i.e., policy ambiguity) on how to implement the policy (e.g., if medication errors were to be reported) and that there was inconsistency in the information being provided to them (e.g., submission of Periodic Safety Update Reports (PSUR)/Periodic Benefit-Risk Evaluation Reports (PBRER) for generic products). This led to companies implementing the policy individually based on their own beliefs on what was required, leading to variations between them.

*”…. the guidelines are not very clear for Kuwait, it’s like all over.”* (Participant 10, pharmaceutical industry, Kuwait).

*“Sometimes I get a question about the PSURs for generics. In the EU [European Union], we don’t have to submit PSURs for generics, but in the Arab guideline it is mandatory. So, who should we follow?”* (Participant 21, pharmaceutical industry, Kuwait).

As was the case with policy ambiguity, participants noted that the nature of the policy also impacted policy conflict. A few participants each from the national centre and the industry in Jordan and Oman as well as many industry participants in Kuwait stressed the importance of the policy’s compliance with both the Arab GVP guideline (and the European GVP guidelines from which it derived), as an important facilitator.

This meant that the goals of the policy were aligned with those of regional and international guidance, which were centred on drug safety and hence decreased the likelihood of conflict occurring due to stakeholders’ differing views.

*“I agree with them [the policy goals], yes, because actually, these are international guidelines. We are not drafting something new. It is all adopted from international guidelines. There are of course certain things that might be customised according to the country, and I agree with them.“ (Participant 10, pharmaceutical industry, Jordan)*.

A few of the national centre participants in Jordan and a few participants each from the national centre and the industry in Oman described how the tailoring of the national policy according to the country’s capabilities also acted as a facilitator for policy implementation by reducing policy conflict around its means of implementation. Participants elaborated that this was done through incorporating only those aspects of the Arab GVP guideline whose implementation was deemed achievable (when considering the local conditions) into the national policy. A few participants from the national centre and some from the industry in Kuwait also cited this factor as acting as a facilitator in reducing conflict associated with policy implementation.

*“I have seen countries who have implemented very vast guidelines, but they don’t know what is in it. So, some countries are… I mean their structure is not capable of implementing those guidelines which already stated to be in place. Whereas in Oman it is not the case, their team have studied [the Arab GVP guidelines] and they have taken only the things that they can implement in this stage.“ (Participant 9, pharmaceutical industry, Oman)*.

#### Implementation planning

Only participants from Jordan (a few each from the national centre and the industry) indicated that decision-makers in the country set up a formal working committee that was tasked with developing the operational aspects of the pharmacovigilance system. Therefore, an implementation plan was laid out whereby it was made clear to implementors how, when, and what aspects of the policy were to be implemented at a particular point in time, and that this facilitated implementation by reducing ambiguity. Although participants in Oman did not indicate the establishment of a formal working committee, a few participants from the national centre and the industry pointed out that there was a constant line of dialogue between the industry and the national centre during the different stages of the implementation process.

*“They [the national pharmacovigilance centre] first of all they started by insisting that you had to have as a company a pharmacovigilance department, not a department as such, but activities, and then you had to have a master file, and then, later on, they said that you should have a resident Omani pharmacist as a local safety person in Oman…”* (Participant 3, pharmaceutical industry, Oman).

Another facilitator described by a few participants from the national centre and many from the industry in Jordan was the national centre carrying out pharmacovigilance inspections, both during the initial stages of the policy implementation and once the policy was fully implemented. This reduced policy ambiguity among industry implementors since it allowed the national centre to not only monitor but also assist with policy implementation.

*“In Jordan, they were able to accompany most of the companies and to provide them with guidance. For a while, maybe a year or two, even when they would say they were coming for an inspection; it was not so much an inspection as it was an assessment of the situation while providing guidance or recommendations…”* (Participant 14, pharmaceutical industry, Jordan).

In contrast, participants from Kuwait agreed that an implementation plan was lacking for the KDFCA, which acted as an implementation barrier by causing ambiguity to companies in terms of how the policy was to be implemented.

*“No [there were no steps taken from the authority with regards to the implementation of the policy], they just issue the policy, and they say, effective so and so date and we have to adhere to that.“* (Participant 10, pharmaceutical industry, Kuwait).

According to a few participants from both sectors in Jordan, a gradual implementation of the policy which involved not mandating policy implementation on all companies (particularly those with little experience in pharmacovigilance) from the outset facilitated implementation. It was explained that a stepwise approach, whereby aspects of the policy which were more achievable (e.g., developing a PSMF and ADR reporting forms) were focused on in the beginning, gradually moving onto more complex aspects (e.g., preparing PSUR/PBRER). This resulted in a smoother implementation process due to reduced conflict between stakeholders around the policy means.

*“…they [the national pharmacovigilance centre] were not tough from the beginning in that they published the guideline today and then required that they be implemented within the next month; they gave the companies sufficient time to have a PSMF, to know how to fill out the form, to adapt the timelines, all of these things.“* (Participant 14, pharmaceutical industry, Jordan).

A few participants from Kuwait’s pharmaceutical industry viewed the national drug authority’s failure to provide them with an adaptation period before implementation, and the lack of an adequate timeframe for proper implementation, as barriers and a source of policy conflict. This contrasted with the situation in Jordan and Oman where participants from both sectors indicated that companies were afforded an adjustment period and a timeframe for policy implementation, thus avoiding policy conflict.

*“In general, in Kuwait, the barriers would be that they impose things without giving a grace period. In other countries, when a new guideline is issued, they inform you that implementation will start from a certain date. They give you a grace period to prepare yourself.“* (Participant 5, pharmaceutical industry, Kuwait).

Despite indicating the presence of an implementation plan, a few participants from Jordan’s national centre, as well as a few from the industry, identified the absence of adequate funding as a barrier. Stakeholder views surrounding policy means were thus incompatible and resulted in policy conflict, which in turn hampered efforts in building awareness and conducting training workshops for stakeholders. In contrast, most participants from Oman and Kuwait did not highlight funding as a factor, indicating the absence of policy conflict.

*“At the end of the day you are in the governmental sector, and in this country, we don’t have resources allocated for pharmacovigilance to promote awareness or other things that we need. We found solutions by forming collaborations with stakeholders, drug manufacturers and drug agents (distributors) to do such events in Jordan.“* (Participant 7, JFDA, Jordan).

#### Company-related factors

A few participants from the pharmaceutical industry in all three countries and a few from the national centre in Oman believed that being a multinational company with experience operating in developed countries, where pharmacovigilance policies and regulations were more stringent acted, as a facilitator. Similarly, a few Jordanian and Kuwaiti industry participants thought that local companies which had licensing agreements with multinational companies facilitated policy implementation due to clauses in their agreements that required standards to be in place on par with those of the multinationals. This meant that there was less ambiguity due to the presence of a degree of familiarity with the guidance and hence policy implementation proceeded more smoothly.

*“…most of the points that are in the [Arab GVP] guidelines it is already implemented by the multinational companies because it is part of the European guideline, so it was easy to implement by these pharmaceutical companies.“* (Participant 7, DGPA&DC, Oman).

A few of Jordan’s and Oman’s pharmaceutical industry participants pointed to the lack of harmonisation among Arab countries in implementing the Arab GVP guideline as part of their national policies. Each country in the region appeared to have its own set of rules and guidelines extracted from the same source, which confused companies operating in multiple countries in the region. This represented a source of conflict between the companies and the national pharmacovigilance centres.

*“Sometimes external regulatory authorities having different requests acts as a barrier. There is a unified guideline, but no unified actions. So, we have the same guideline, but different requests, regulations in each country.“* (Participant 15, pharmaceutical industry, Jordan).

**Table. 4:** Factors impacting policy ambiguity and conflict

	Ambiguity			Conflict		
	**Jordan**	**Oman**	**Kuwait**	**Jordan**	**Oman**	**Kuwait**
**Political support**	Decrease	Decrease	Increase	Decrease	Decrease	Increase
**Stakeholder involvement**	Decrease	Decrease	Increase	Decrease	Decrease	Increase
**Training**	Decrease	Decrease	Increase	N/A	N/A	N/A
**Policy characteristics**	Decrease	Decrease	Increase	Decrease	Decrease	Decrease
**Implementation planning**	Decrease	Decrease	Decrease	Decrease	Mix	Mix
**Company-related factors**	Decrease	Decrease	Decrease	Increase	Increase	Increase

## Discussion

This is the first study to employ Matland’s [28] ambiguity-conflict model of policy implementation to analyse and identify the type of pharmacovigilance policy implementation process in three Arab countries with differing levels of system performance (Jordan, Oman, and Kuwait). This in turn is used to inform recommendations for the implementation of a pharmacovigilance policy (incorporating the Arab GVP guideline) in countries with pharmacovigilance systems at a nascent stage of development (such as Kuwait). The qualitative approach based on interviews allowed for gaining a deep understanding of the mechanisms as well as the facilitators and barriers to pharmacovigilance policy implementation in Arab countries. Application of the two dimensions of Matland’s model (i.e., the levels of policy ambiguity and policy conflict in its development and implementation) provided a novel yet manageable approach to identifying the process and factors associated with successful pharmacovigilance policy implementation.

This study suggests that the elements underlying successful policy implementation in Jordan and Oman were rooted in their respective approaches and include management support, policy characteristics (including realistic policy objectives as well as clarity of policy goals and means) stakeholder involvement, training, and implementation planning. In contrast, these elements were either absent or not properly considered in Kuwait. These could be the underlying reasons for its lagging pharmacovigilance system in comparison with other Arab countries. In what follows, each of these elements will be discussed in the context of Matland’s [28] model and other existing literature on policy implementation.

Effective management support has been demonstrated to motivate implementors to carry out their functions, whereas a lack of engagement by senior officials causes implementors to feel isolated and insecure. [39] Additionally, management support aids in the elimination of structural obstacles conflicting with successful policy implementation such as resource shortages. [40] In Matland’s [28] model, these two aspects of management support are related to both reducing policy ambiguity and conflict. In Jordan and Oman, decision-makers provided support throughout the entire process by initiating and guiding the policy’s implementation. These actions reduced policy ambiguity and conflict by ensuring clarity and agreement regarding policy goals and means of achievement respectively. In contrast, management support was missing from Kuwait’s pharmacovigilance policy process thus acting as an impediment that caused policy ambiguity to implementors regarding their roles. This needs to change to motivate implementors to follow through with implementation.

Governments of developing countries often devise policies with ambitious goals without considering the practicality of implementing them given the local contextual factors. This results in an implementation gap with many policy goals left unfulfilled. [41] This issue relates to policy conflict within Matland’s [28] model which arises due to differences in stakeholder’s views regarding how the policy goals are to be met.

The Arab GVP guideline was designed as a model of best practice to be followed by countries in the region. However, it is flexible in allowing the individual countries to implement the parts that suit them at the time and based on the available resources and capacities. [23] Jordan and Oman’s policies benefitted from this flexibility by focusing on aspects that could be practically implemented given their respective capacities thus allowing for a smoother implementation process. This approach was also followed, though to a lesser extent, in Kuwait. Therefore, it should be given greater consideration by its decision-makers when implementing future iterations of the policy.

Studies have shown that policy clarity is a significant factor affecting policy implementation. [35, 37, 42, 43] This is consistent with Matland’s [28] model which relates policy ambiguity to the clarity of its goals and the impact of local conditions on implementation. A distinct feature of Jordan and Oman’s policies was their simplicity and attention to detail leading to low ambiguity thus making it easy for implementors to understand what was required of them. Kuwait’s policy was not sufficiently clear in delineating the roles and responsibilities of each side, which is consistent with high ambiguity in Matland’s [28] model. This resulted in differences in implementation among companies. Policymakers in other Arab countries with nascent systems would be wise to learn from these experiences by developing a policy that clearly outlines the roles and responsibilities of all parties involved in the implementation process.

Stakeholder involvement in the policy development and implementation process has been identified as an important means of ensuring a sense of ownership of the policy. [44, 45] In Jordan and Oman, the active involvement of members of the national centre and the pharmaceutical industry in developing and implementing the pharmacovigilance policy contributed to a better understanding of its practical implementation (i.e., low ambiguity). Moreover, agreement of both sides meant less opposition (i.e., low conflict) during policy implementation. However, in Kuwait, pharmaceutical companies were not involved at any stage of the policy process. Consequently, pharmaceutical companies did not fully understand their responsibilities regarding the pharmacovigilance policy’s implementation and thus viewed it as highly ambiguous.

Given the important role human resources play in the policy implementation process, ensuring proper training and orientation regarding the policy becomes a priority. [45] Properly trained policy implementors possess greater competency and self-belief to overcome obstacles that they may face. [43, 46, 47] In Jordan and Oman, policy implementors from both sectors underwent training thus facilitating implementation by ensuring that all involved parties understood their roles and responsibilities (i.e., low ambiguity). In Kuwait, however, the absence of training related to the policy meant that implementors (particularly from the industry) lacked knowledge regarding the policy (i.e., high ambiguity). Decision-makers in Kuwait, as well as in other counties with nascent systems, should adopt training programmes in the future when implementing policies, especially complex policies which encompass a large group of people.

The presence of a strategic plan for the implementation process, which includes priorities, goals, and timelines, is an important prerequisite for successful policy implementation. [48] Furthermore, for a policy to be fully implemented, sufficient time is required, which is often underestimated by policymakers. [49] The highest level of policy implementation planning was observed in Jordan, while at the other end of the spectrum was Kuwait, where setting an implementation plan seemed to be neglected. Moreover, it was observed that Jordan and Oman’s pharmaceutical companies were provided appropriate implementation timeframes, which decreased policy conflict thus facilitating proper policy implementation. The lack of a comparable implementation timetable in Kuwait meant that implementation in some companies was delayed.

In implementing health-related policies, the WHO recommends proceeding in a stepwise manner starting with the easier aspects of the policy to ensure high visibility, success, and support during the initial stages. [50] Policy implementation planning in Oman and Jordan embodied a stepwise approach in implementation whereby the policy’s simpler aspects were required to be implemented first and the more complex aspects implemented gradually thereafter. This resulted in lower policy ambiguity and conflict. This was absent in Kuwait as all aspects of the policy were implemented in a single phase resulting in confusion (i.e., high ambiguity) and subsequent delays in some companies’ implementation. This evidence suggests that a stepwise approach be taken into consideration by other countries with nascent systems in their implementation planning of a pharmacovigilance policy.

Jordan’s pharmacovigilance policy allowed for monitoring, evaluating, and enforcing policy implementation by conducting inspections of companies’ pharmacovigilance systems and processes. This served as a tool for the national centre to positively educate pharmaceutical companies on the proper implementation of the policy. Whilst Oman’s policy did contain provisions that would allow pharmacovigilance inspections to be undertaken in the future, this tool was not available in Kuwait. Therefore, these countries’ national centres were not able to evaluate companies’ implementation of the policy and take corrective actions as required. The presence of such a mechanism permits continuous progress assessment, provides transparency as well as accountability and serves as a means of comparison across locations and time. [50] Moreover, it serves as a means of obtaining feedback on policy implementation progress, which permits policymakers to make the necessary adjustments as needed. [26] This points to policy implementation planning having a role in the reduction of policy ambiguity and conflict.

Applying Matland’s [28] model to the factors impacting the pharmacovigilance implementation process in conjunction with participants’ views on its ambiguity and conflict enabled the discernment of policy ambiguity and conflict levels within the three countries, and the type of implementation strategy being followed. The presence of both low policy ambiguity and conflict in Jordan and Oman points to the presence of a structured approach to policy development and implementation. This suggests that the implementation process’ characteristics in these countries were consistent with what Matland [28] describes as “administrative implementation”. Given that the implementors were clear about and supportive of the goals of the policy in this type of implementation, the primary strategy becomes ensuring that adequate resources are provided by those at the top. [28] In Kuwait however, while participants’ views and the cited factors impacting policy point to high ambiguity, there were differences in terms of policy conflict. On the one hand, participants’ views surrounding policy conflict pointed to low conflict, while on the other hand, the cited factors’ impact pointed to an increase in conflict. This suggests policy implementation falling in between the “experimental implementation” and “symbolic implementation” processes. In both cases, success is variable across locations and is dependent on contextual factors such as the actors involved and resource availability. However, in the former, the process’ focus is on learning about policy impacts. [28] While in the latter, successful outcomes are often “determined by the coalition of actors at the local level who control the available resources” (28, p. 168). Both mechanisms are consistent with the differences in policy implementation by companies in Kuwait due to it occurring based on each companies’ understanding. Therefore, it is recommended that policymakers follow a more structured process in developing and implementing pharmacovigilance policy to reduce ambiguity and conflict, thus moving in the direction of “administrative implementation”.

This study has a few limitations. First, there was potential for response bias to occur due to interviewees’ hesitation of criticising decision-makers, which was mitigated by assuring participants of their anonymity and the confidentiality of their views. Second, participants’ responses could have been exaggerated or contain inaccuracies because they were reliant on memory. This was minimised by confirming information from more than one participant, comparison with information from the literature and correspondence with the participants for clarification purposes. Finally, the views of healthcare professionals or patients were not explored.

A major strength of the study lies in its use of a theoretical framework adapted from policy implementation research to guide the study. The study’s use of a qualitative approach is also a strength as it provided detailed information on the policy implementation process in the three study countries which allowed for comparison between them. Finally, the inclusion of members of the pharmaceutical industry provided an additional perspective on the issues concerning pharmacovigilance policy implementation.

## Conclusions

Threats posed by ADRs necessitate the implementation of policies aimed at safeguarding public health. Based on the study findings, several recommendations are put forward to reduce policy ambiguity and conflict and facilitate an orderly implementation process:

- Strengthening decision-makers’ relationships with implementors through continuous engagement and communication to provide guidance and motivation.
- Ensure that all relevant stakeholders (including health authority, industry, and healthcare professionals) are involved in the policy development and implementation processes whereby their feedback is taken into consideration.
- Carry out awareness campaigns and training workshops targeted at all relevant stakeholders to increase knowledge and understanding regarding the policy’s goals and means of implementation.
- Develop a tailored pharmacovigilance policy that is in line with available capacities and capabilities.
- Ensure that the policy clearly outlines the roles and responsibilities of all stakeholders involved in the implementation process and binding it into law.
- Planning should be carried out prior to policy implementation whereby the process’ needs in terms of time and resources are assessed, objectives and milestones with suitable timeframes are outlined, and suitable adjustment periods are provided.
- Ensure that policy implementation follows a stepwise approach that is gradual whereby, as time passes, the aspects of the policy that are implemented increase in their level of complexity.
- Have in place a process for monitoring and evaluating policy implementation consistency, accuracy, and compliance which is non-punitive.

The lessons learned from studying the three countries can help guide both the development and implementation of pharmacovigilance policy in other countries with nascent pharmacovigilance systems and move countries in the region closer towards their shared goal of harmonisation based on the Arab GVP guideline. Future research could focus on the views of other stakeholder groups including healthcare professionals, academics, and patients regarding the pharmacovigilance policy and its implementation.

## Data Availability

The datasets used and/or analysed during the current study are available from the corresponding author on reasonable request.

## Abbreviations

ADR: Adverse drug reaction
DGPA&DC: Directorate General of Pharmaceutical Affairs and Drug Control
EU: European Union
GVP: Good Pharmacovigilance Practices
JFDA: Jordan Food and Drug Administration
KDFCA: Kuwait Drug and Food Control Administration
MOH: Ministry of Health
PBRER: Periodic Benefit-Risk Evaluation Report
PSMF: Pharmacovigilance System Master File
PSUR: Periodic Safety Update Report
WHO: World Health Organization
